# Nitrogen loss by anaerobic ammonium oxidation in unconfined aquifer soils

**DOI:** 10.1038/srep40173

**Published:** 2017-01-10

**Authors:** Shanyun Wang, Dirk Radny, Shuangbing Huang, Linjie Zhuang, Siyan Zhao, Michael Berg, Mike S. M. Jetten, Guibing Zhu

**Affiliations:** 1 Key Laboratory of Drinking Water Science and Technology, Research Center for Eco-Environmental Sciences, Chinese Academy of Sciences, Beijing, China; 2 Eawag, Swiss Federal Institute of Aquatic Science and Technology, 8600 Dubendorf, Switzerland; 3 Institute of Hydrogeology and Environmental Geology, Chinese Academy of Geological Sciences, Shijiazhuang 050061, China; 4 Department of Microbiology, Radboud University, Nijmegen, Netherlands; 5 Department of Biogeochemistry, Max Planck Institute for Marine Microbiology, Bremen, Germany

## Abstract

Anaerobic ammonium oxidation (anammox) is recognized as an important process for nitrogen cycling, yet little is known about its role in the subsurface biosphere. In this study, we investigated the presence, abundance, and role of anammox bacteria in upland soil cores from Tianjin, China (20 m depth) and Basel, Switzerland (10 m depth), using isotope-tracing techniques, (q)PCR assays, and 16 S rRNA & *hzs*B gene clone libraries, along with nutrient profiles of soil core samples. Anammox in the phreatic (water-saturated) zone contributed to 37.5–67.6% of the N-loss (up to 0.675 gN m^−2 ^d^−1^), with anammox activities of 0.005–0.74 nmolN g^−1 ^soil h^−1^, which were even higher than the denitrification rates. By contrast, no significant anammox was measured in the vadose zone. Higher anammox bacterial cell densities were observed (0.75–1.4 × 10^7 ^copies g^−1 ^soil) in the phreatic zone, where ammonia-oxidizing bacteria (AOB) maybe the major source of nitrite for anammox bacteria. The anammox bacterial cells in soils of the vadose zone were all <10^3 ^copies g^−1 ^soil. We suggest that the subsurface provides a favorable niche for anammox bacteria whose contribution to N cycling and groundwater nitrate removal seems considerably larger than previously known.

Nitrogen (N) is an essential nutrient for all organisms, and it is fundamental to the biochemical processes that define life. The global N cycle has historically been considered a “linear” process: N in the form of atmospheric nitrogen gas (N_2_) is fixed and transferred as ammonium (NH_4_^+^) to the Earth’s surface, oxidized to nitrate (NO_3_^−^), and finally reduced and returned back to the atmosphere as N_2 _gas. The various steps in this process are mediated by microbial activity^1^. Much has been learned about these steps and many of the microorganisms involved^2^,^3^. In today’s consideration of the N cycle, the surface ecosystem is still emphasized, while much less attention is paid to the subsurface biosphere^4^,^5^.

For decades, the conversion of fixed nitrogen to dinitrogen gas by heterotrophic bacteria, termed heterotrophic denitrification, was considered the main pathway of N loss in natural ecosystems^6^. The discovery of anaerobic ammonium oxidation (anammox) mediated by autotrophic anammox bacteria, which were able to oxidize ammonia directly to N_2_ without nitrous oxide (N_2_O) emission, greatly changed our view of the marine and terrestrial N cycles^7^,^8^,^9^. Anammox has been detected worldwide in natural ecosystems. In marine systems, it may be responsible for up to 50% of the marine N loss^10^,^11^, with hotspots occurring in oxygen minimum zones (OMZ)^12^,^13^,^14^. Anammox has also been detected in many freshwater systems with hotspots occurring in riparian sediments^15^,^16^,^17^,^18^. Overall, the available data indicates that anammox may be present in many environments, including surface aquatic ecosystems, and that anammox may be responsible for a significant proportion of N_2_ production. However, the prevalence of anammox in upland fields has not been identified yet^19^. We speculated that anammox would not occur significantly in the surface soil system but would occur in the subsurface biosphere, especially in the phreatic groundwater-saturated subsurface^20^,^21^.

Aquifer soils include soils in the vadose, phreatic, and aquitard zones. The phreatic zone stores groundwater, which plays a central part in drinking water production, agricultural irrigation, ecosystem sustenance, human adaptation to climate change, and global food security^22^,^23^. The contribution of anammox to N loss in groundwater was first observed in Massachusetts, USA using isotope tracers^24^. Subsequently, anammox in groundwater was investigated at several sites, such as Mansfield, UK^25^, Waterloo, Canada^26^, and in an *in situ* experiment at Cape Cod, MA, USA^27^ where the activity and contribution of anammox varied significantly due to groundwater mobility and allochthonous pollution. Interestingly, anammox in aquifer soils has not been examined to date. Prior studies have not determined whether anammox occurs only in fluent groundwater or whether it occurs in local aquifer soils, where the mechanism of anammox N loss appears different because of the interacting forms.

The aim of the present study was therefore to investigate the distribution, activity, contribution and microbial mechanism of anammox in soil profiles that represent both the vadose water-unsaturated zone and the phreatic zone of two unconfined aquifers in China and in Switzerland, in order to determine whether anammox is significant in subsurface environments. In addition, the potential interspecies relationships among anammox, nitrifiers (ammonia-oxidazing bacteria (AOB) and archaea (AOA)), and even denitrifying anoxic methane oxidizing population (n-DAMO, the fourth oxygen-producing biological pathway^28^) were also analyzed to find out whether these microorganism could form a novel pathway of anammox synergism in the subsurface biosphere. In this study, the term “aquifer” is defined as the phreatic water-saturated zone below the groundwater table of an unconfined aquifer, while the term “non-aquifer” is used to describe the vadose water-unsaturated zone above the groundwater table.

## Results

### Identification and quantification of anammox bacterial abundance in soil cores

**Figure 1:**
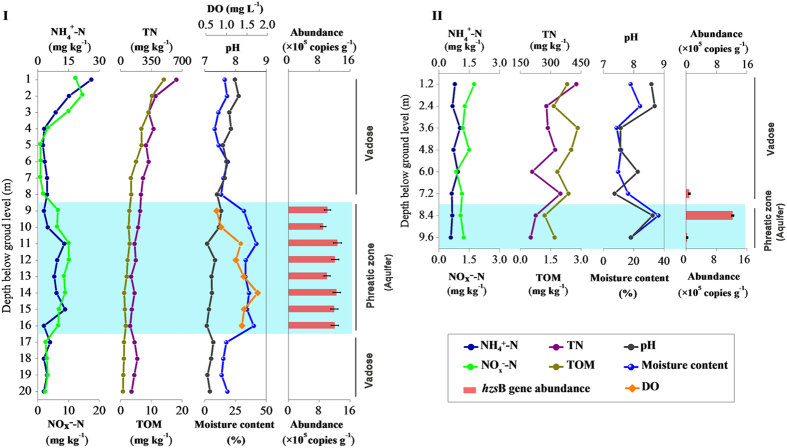
The vertical distribution of ammonium, NO_x_^−^, total Kjeldahl nitrogen, total organic matter, pH, water content, and anammox bacterial abundance in deep soil cores for Tianjin (I) and Basel (II) aquifers. The aquifer zone is indicated in light blue.

For the Tianjin and Basel core samples, the aquifers were located at a depth of 8–16 m and less than 7.2 m, respectively. The aquifer in Tianjin had characteristics of ammonium and nitrate pollution, containing up to 8.9 and 15.1 mg N kg^−1^, respectively. The NH_4_^+^ and NO_3_^−^ concentrations in the Basel soil samples were all below 2 mg N kg^−1^ (Fig. 1). The presence of anammox bacteria was established by amplification of the *hzs*B and 16 S rRNA gene using primers specific for anammox. The positive PCR products of anammox bacterial gene were only obtained in aquifer soils. Samples from other depths generated no positive PCR results. The BLAST analysis of the gene sequences confirmed that all the clones represented anammox-like sequences.

The anammox bacteria abundance was then estimated with qPCR targeting the *hzs*B gene, which is diagnostic for anammox bacteria. In Tianjin soil cores, the anammox numbers were all below 10^3^ copies g^−1^ above the aquifer. A significant peak of anammox was detected in the aquifer (8–16 m bgl) where abundance ranged from 9.0 × 10^5^ to 1.3 × 10^6^ copies g^−1^ dry soil. Below the aquifer, the anammox abundance decreased to the detectable level (~10^3 ^copies g^−1^).

The same trend was obtained in the Basel soil core. The peak of anammox bacteria was only distributed in the aquifer (7.2 m bgl) and ranged from 3 × 10^4^ to 1.2 × 10^6 ^copies g^−1^ dry soil. At other depths, no anammox abundance was detectable.

### Activities, contribution, and role of anammox in the soil cores

**Figure 2:**
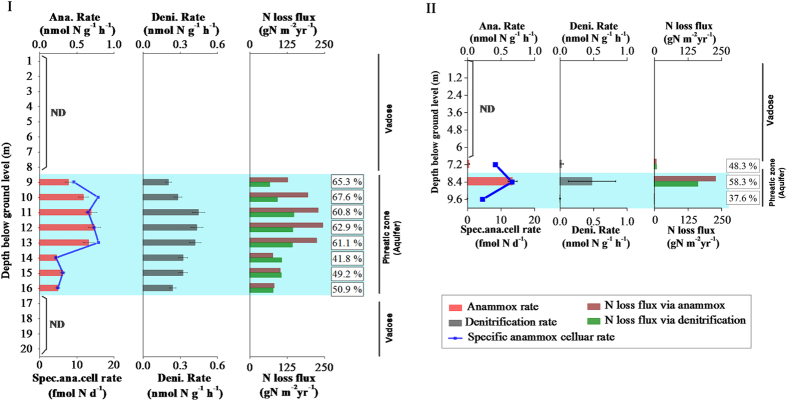
The vertical distribution of anammox bacterial rate, specific anammox cellular activity, denitrification rate, and the contribution of anammox and denitrification for N loss in soil cores for Tianjin (I) and Basel (II) aquifers. The aquifer zone is indicated in light blue. ‘ND’ indicates the abundance of anammox below the detection limit (<10^3 ^copies g^−1^), meanwhile, no positive rate of anammox was detected.

The anammox activity and the potential role of anammox as a source of N loss in the soil cores were determined by performing incubations with homogenized soil under *in situ* temperatures using a ^15^N-labeled isotope-tracing technique. The depth profiles of Tianjin upland soils containing activated anammox were only in aquifer soils at rates of 0.23–0.74 nmol N g^−1 ^h^−1^ (Fig. 2I), which were in agreement with molecular results. Strikingly, some samples showed anammox rates for N loss that were even higher than those of denitrification (*n* = 5). The specific cellular anammox activities were then calculated based on total anammox activity and abundance. In the Tianjin aquifer, the specific anammox activity ranged from 4.5–16.0 fmol cell^−1 ^d^−1^ based on analysis of the anammox *hzs*B gene^9^, and this activity is at the upper end of reported values (2–20 fmol cell^−1 ^d^−1^ refs 8 and 29). Here, moisture content of soil samples seemed to be the key positive factor influencing anammox rates (*r* = 0.844, *p* = 0.000, Supplementary Table S2). The other samples of the Tianjin soil profile showed no significant anammox activity. In the Basel aquifer, the measured anammox ranged from 0.005–0.68 nmol N g^−1 ^h^−1^ with 37.5–58.3% N loss (Fig. 2II). At other depths, no significant anammox activity was measured. The specific activity ranged from 4.5–13.3 fmol cell^−1 ^d^−1^. These data indicate that denitrification is not the only significant pathway for N loss in the aquifer. In the Basel aquifer samples, the water content also had a significant positive correlation with the anammox rate (*r* = 0.826, *p* = 0.000, Supplementary Table S3).

Based on the anammox rates and bulk density of all soils (2.6–2.8 g cm^−3^), the N loss flux attributed to the anammox in the aquifers was estimated. In the soil core samples from the Tianjin aquifer, the N loss via anammox was 0.214–0.675 g N m^−2 ^d^−1^, while the contribution of denitrification to the N loss was 0.187–0.408 g N m^−2 ^d^−1^. It indicated that the anammox contributed a significant percentage (41–67%) to microbial N_2_ production and N removal in the aquifer. In the soil core samples from the Basel site, the N loss via anammox was 0.005–0.634 g N m^−2 ^d^−1^, while the contribution of denitrification to the N loss was 0.008–0.453 g N m^−2 ^d^−1^. These data indicate that anammox is an important but overlooked alternative process to denitrification for microbial N loss.

### Biodiversity and community of anammox bacteria in the aquifers

**Figure 3:**
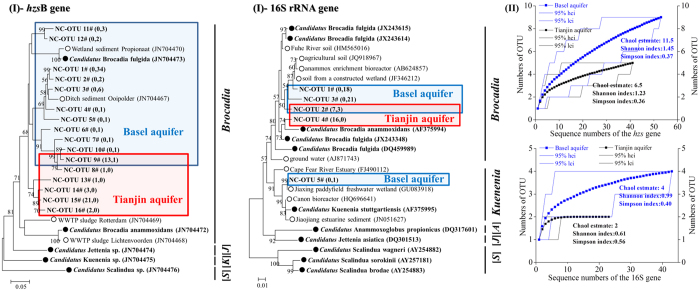
Phylogenetic and rarefaction analysis of anammox bacterial communities using the *hzs*B gene and 16 S rRNA gene at the Tianjin and Basel aquifer zones. An evolutionary distance dendrogram (constructed by the neighbor-joining method using the maximum composite likelihood distance with a 1,000 bootstrap in a MEGA 4.0 software package) shows the affiliations of the anammox bacterial *hzs*B gene sequences (above) and the 16 S rRNA gene sequences (below) retrieved from the aquifer soils in Tianjin and Basel. The OTUs are identified in bold with the number of sequences identified in brackets from the Tianjin aquifer (41 *hzs*B and 23 16 S rRNA gene sequences in total) and the Basel aquifer (53 *hzs*B and 43 16 S rRNA gene sequences in total). The known anammox bacterial sequences are identified with solid circles. The designators [*J*], [*K*], [*S*], and [*A*] indicate the anammox bacteria “Jettenia,” “Kuenenia,” “Scalindua,” and “Anammoxoglobus,” respectively. The details for the anammox clone sequences of each OTU and for the accession numbers in the Genbank are listed in Supplementary Tables S3 and S4. The DOTUR program was used with 2% sequence variation (for *hzs*B gene sequences) and 3% sequence variation (for 16 S rRNA gene sequences) for OTU determination.

The biodiversity and community composition of anammox bacteria in the aquifers were investigated by analyzing the *hzs*B and 16 S rRNA gene sequences retrieved from clone libraries using rarefaction analysis, the Chao1 estimator, and Shannon index calculations (Fig. 3). Phylogenetic analyses of anammox bacterial *hzs*B and 16 S rRNA gene sequences and related sequences deposited in the GenBank showed that all sequences obtained from soils from the Basel aquifer and Tianjin aquifer were most closely related to “*Candidatus* Brocadia” (similarity 94.6–97.6%) (Fig. 3I). The analyses of the *hzs*B and 16 S rRNA gene sequences showed that a total of 10 and 4 OTUs, respectively, were obtained from the Basel aquifer based on the *hzs*B (98% cut-off) and 16 S rRNA gene sequences (97% cut-off). Nevertheless, the anammox bacteria from the Tianjin aquifer showed a very limited anammox bacterial diversity with a total of 6 and 2 OTUs respectively obtained for the *hzs*B and 16 S rRNA gene sequences. The *hzs*B and 16 S rRNA gene sequences both showed a higher biodiversity in soil samples from the Basel aquifer (with little human activity and good groundwater quality) than from the Tianjin aquifer (Fig. 3II).

### Quantitative analysis of related N cycle processes with anammox in aquifer soils

**Figure 4:**
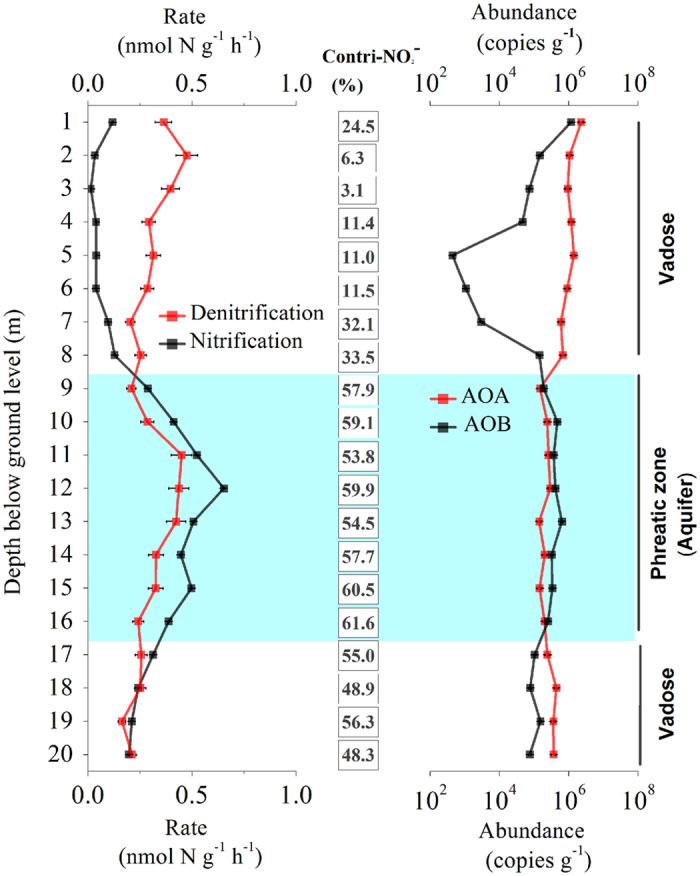
The vertical distribution of nitrification and denitrification activities and the relevant archaeal and bacterial *amo*A gene copy numbers retrieved from deeper soils (20 m bgl) of the Tianjin aquifer. The percentages of nitrification to denitrification and ammonia-oxidizing archaea to ammonia-oxidizing bacteria are shown in the right-hand columns of panel a and panel b, respectively.

The biogeochemical mechanism of anammox in the aquifer was investigated in the Tianjin soil cores, and the potential rates of nitrification and denitrification were determined to assess the source of nitrite for anammox. The nitrification and denitrification rates decreased from the surface to the aquifer, and denitrification rates (0.2–0.47 nmol N g^−1 ^h^−1^) were all higher than nitrification rates (0.02–0.13 nmol N g^−1 ^h^−1^) in the vadose zone (Fig. 4). This situation was completely reversed in the aquifer, where both the nitrification and denitrification rates increased, and nitrification rates (0.29–0.66 nmol N g^−1 ^h^−1^) were all higher than denitrification rates (0.21–0.45 nmol N g^−1 ^h^−1^). Therefore, in the aquifer, both partial denitrification and nitrification may provide anammox with nitrite, but nitrification may be the main nitrite source for anammox.

**Figure 5:**
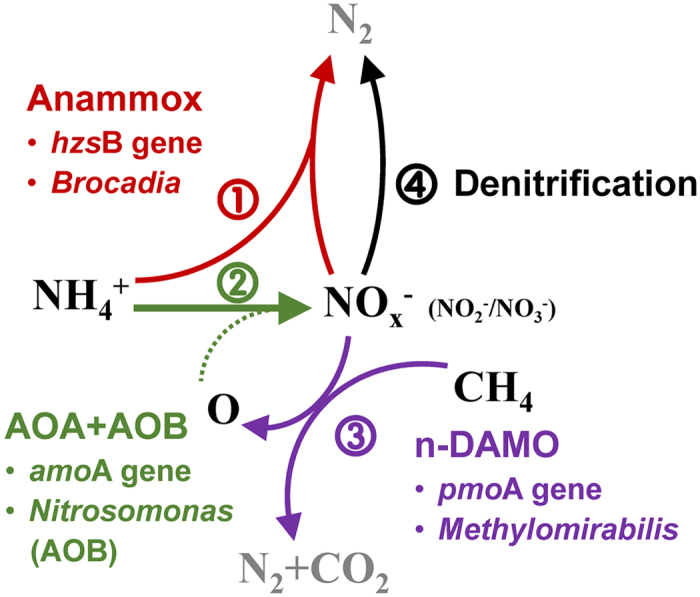
The potential interspecies relationships among anammox bacteria, AOB&AOA, n-DAMO and denitrification, and the related dominated genera of anammox bacteria, AOB&AOA, and n-DAMO, respectively. The affiliation of AOA&AOB *amo*A gene and n-DAMO *pmo*A gene sequences were shown in Supplementary Figure S3.

The potential of a combined nitrification–anammox process in aquifer soils was further explored by performing qPCR assays on bacterial and archaeal *amo*A genes in the soil core (0–20 m). The qPCR data for the soils above the aquifer showed higher gene numbers for the archaeal *amo*A gene (6.7 × 10^4^–2.2 × 10^6^ copies g^−1^ dry soil) than for the bacterial *amo*A copies (2.6 × 10^3^–1.1 × 10^6 ^copies g^−1^ dry soil). However, in the aquifer, the abundance was higher for AOB (4.4 × 10^4^–9.1 × 10^4 ^copies g^−1^) than for AOA (7.1 × 10^3^–2.8 × 10^4 ^copies g^−1^ dry soil). Multiple linear stepwise regression analysis also identified the AOB abundance to be the most determining variable regarding nitrification activity (Supplementary Table S5). Furthermore, the n-DAMO bacteria, which conduct the fourth biological pathway that produces oxygen^28^, were also detected in the samples where AOA, AOB, and anammox bacteria were present. A potential interaction among AOA (‘*Nitrososphaera*’ dominant), AOB (‘*Nitrosomonas*’ dominant), n-DAMO (‘*Methylomirabilis*’), and anammox bacteria (*Brocadia*) would form a new pathway of anammox synergism in the subsurface biosphere (Fig. 5, Supplementary Figure S4).

## Discussion

In the present study, the activity, contribution, and role of anammox in subsurface deep upland soils were investigated. Results showed that anammox spieces in the upland soil cores were low biodiversity but highly region-heterogeneous. Below the groundwater level, anammox bacterial abundance and activity were detected at all depths of each soil core. In contrast, above the groundwater level, anammox bacterial abundance was below the detection limit (~10^3 ^copies g^−1^). Anammox in the phreatic zone contributed 37.5–67.6% of the N loss with activities of 0.005–0.74 nmol N g^−1 ^soil h^−1^ that were even higher than the denitrification rates (0.337 ± 0.092 nmol N g^−1 ^soil h^−1^, *t* - test, *p* = 0.000). The discovery indicate that anammox process actually occurred in local aquifer soils, and significantly contribute in subsurface soil biospheres, especially in the aquifers zone.

The anammox process showed distinct biogeochemical features in aquifer soils and in groundwater. Anammox activity has been reported in groundwater at several sites^24^,^27^,^29^,^30^. Due to groundwater mobility and allochthonous pollution, the activity and contribution of anammox in groundwater were significantly different at each site. Böhlke *et al*.^24^ first detected anammox in groundwater with isotope tracers, with a rate of up to 0.027 μmol L^−1 ^d^−1^, indicating that anammox played only a minor role in the N cycle. Clark *et al*.^26^ also provided ^15^N evidence for anammox in anoxic groundwater, and Moore *et al*.^30^ reported bacterial activity of anammox at study sites where groundwater was contaminated by ammonium, with a contribution of 18–36% N loss. In a recent report by Smith *et al*.^25^, anammox was found to contribute 39−90% of potential N_2_ production in wastewater-derived contaminated groundwater. These results suggest the possibility of site-specific heterogeneity in anammox bacterial distributions in groundwater systems. In contrast to the fluidity of groundwater, aquifer soils were stationary, and anammox in aquifers also showed much less fluctuations. In Tianjin aquifer soils, anammox rates were stable at 0.49 ± 0.21 nmol N g^−1 ^soil h^−1^ (*t* - test, *p* = 0.000) and contributed 41−67% of the potential N_2_ production with little variation.

While a high anammox bacterial diversity was reported for groundwater, the aquifers soils of our study exhibited an almost uniform pattern. Anammox bacteria, “*Candidatus* Brocadia anammoxidans” and “*Candidatus* Kuenenia stuttgartiensis,” were detected at an ammonium-contaminated site in Mansfield, UK^31^. In wastewater-derived contaminated groundwater (USA), Brocadia-like bacteria and “*Ca*. Kuenenia spp.” were also detected^27^. Most interestingly, in the ammonium-contaminated aquifer, four of the five known genera of anammox sequences were present, i.e. “*Ca*. Scalindua,” “*Ca*. Jettenia,” “*Ca*. Kuenenia,” and “*Ca*. Brocadia” genus^30^. Yet in aquifer soils, only one genus, “*Candidatus* Brocadia,” was identified in both Tianjin and Basel aquifer soils based on phylogenetic analysis of the anammox bacterial 16 S rRNA and *hzs*B genes. The anammox bacterial biodiversity in the subsurface biosphere was significantly different from that commonly found in the surface biosphere. In surface aquatic systems, the anammox bacteria consistently showed a very low biodiversity^11^,^32^. For example, only bacteria of the “Scalindua” genus were detected in marine ecosystems, while “*Candidatus* Scalindua brodae”’ and “*Candidatus* Brocadia anammoxidans” were detected in Lake Tanganyika^33^ and river sediments^17^, respectively. In wastewater treatment systems, the biodiversity was also low^34^. In contrast, different anammox genera co-existed in various wetland soils ecosystems. In paddy field soils, four genera of anammox bacteria, “Brocadia,” “Kuenenia,” “Anammoxoglobus,” and “Jettenia” were detected^21^. In addition, a high biodiversity was observed in various soil ecosystems, such as peat soil, permafrost soil, and agricultural soil^19^,^35^. Hence, the mechanism of anammox N loss in local aquifer soil appears to be different from that of fluent groundwater and needs further investigation.

In contrast to numerous reports on the widespread distribution of anammox in aquatic ecosystems, there has been no report about the prevalence of anammox in surface upland fields^19^ until now. Hence, in the present study, we investigated anammox in deep upland soils. Both the molecular and isotope results showed that anammox did not occur in upland surface soils, but was only mediated in water logged aquifer soils. In aquifer soils, the anammox abundance (above 10^5 ^copies g^−1^ soil) and rate (above 0.01 nmol N g^−1 ^soil h^−1^) were significantly above the detection limit compared with the non-aquifer zone where anammox bacterial abundance and rate were undetectable (below 10^3 ^copies g^−1 ^soil). In the Tianjin aquifer, the increases in NH_4_^+^ and NO_x_^−^ were considerably higher than those in non-aquifer soils and were also higher than those in Basel soils. However, the biogeochemical correlation analysis showed that the substrate (ammonia or nitrate) had little positive influence on anammox rates in either the Tianjin or Basel aquifers. In the Basel aquifer, by contrast, the NH_4_^+^ and NO_x_^−^ levels were as low as 1–2 mg kg^−1^, and were at the same levels as in the non- aquifer samples. Biogeochemical correlation analysis showed that the water contents had the most positive influence on anammox abundance in the Basel aquifer (*r* = 0.977, *p* = 0.000). Therefore, the substrate contents may not be the key limiting factors for anammox, while water contents showed a positive relation with anammox occurrence. Our previous studies demonstrated that ubiquitous anammox occurs in freshwater at various levels of substrate concentration^18^, indicating that water content controls are important for the anammox reactions. Previous studies, including ours, have clearly shown that abundant anammox bacterial cells exist in upland soils after flooding where no anammox bacteria were detected before flooding^18^,^36^. It may due to higher material mobility and electron transport caused by water. We speculated, therefore, that as the direct substrate of anammox bacteria, ammonia and nitrate contents in soils were the most influential factors determining anammox rate, whereas the water content was the key factor controlling anammox occurrence. After oxygen was consumed in the water-limited diffusion process, an potential anoxic niche for anammox bacteria was created. This was likely responsible for the observed patterns of occurrence, which need to be further investigated.

The discovery of anammox in aquifer soils provides a new perspective on the N cycle and groundwater N pollution. Since the 1970 s, NO_3_^−^ contamination (>10 mg L^−1^, USA EPA) of groundwater has become a significant environmental problem with many parts of the world now reporting groundwater nitrate pollution^37^. Denitrification has been emphasized as the dominant nitrate attenuation process in the phreatic water-saturated zone with minor roles played by other nitrate depletion mechanisms, such as dissimilatory nitrate reduction to ammonium (DNRA), assimilation of nitrate into microbial biomass, and nitrate removal via phreatophytes^38^. In the present study, our use of isotopic tracing technology revealed that up to 37.5–67.6% of aquifer soil N_2_ production, with rates of 0.005–0.74 nmol N g^−1 ^soil h^−1^, was contributed by anammox. At some depths in the aquifer soils, we found that anammox contributed even more than denitrification. The different metabolic pathways in anammox and denitrifying bacteria may give rise to different contributions of anammox and denitrification to nitrate removal. Denitrifiers were mostly facultative anaerobic heterotrophs, which obtain both their energy and carbon from the oxidation of organic compounds^39^. Oxic aquifers often contain low concentrations of dissolved organic matter^37^, limiting the energy needed by denitrifying bacteria that use organic carbon as the electron donor. However, the anammox process is catalyzed by the autotrophic anammox bacteria^40^. Hence, when organic compounds were limited, the anammox bacteria were favored, as has been widely documented in anammox bioreactor research^3^,^20^,^39^. An estimated 0.005–0.675 g N m^−3 ^d^−1^ was lost via anammox in the aquifer soils, indicating that the anammox process is an effective and feasible way to counteract nitrate pollution in groundwater.

Potential interactions have been suggested between particle-associated anammox bacteria and archaeal partners with n-DAMO bacteria in the deep soils. In these oxygen-limited aggregates, cooperation between AOA and AOB can provide nitrite to anammox bacteria^39^,^41^ in a partnership similar to those reported for marine snow particles^13^,^14^,^42^. Simultaneous nitrite-dependent anaerobic methane and ammonium oxidation processes have also been reported in various bioreactors with efficient nitrogen removal loadings and rates^43^,^44^. Although the oxygen production rates by damo bacteria were still not available, the affinity (*Ks*) value of AOA was very low (133 nM^45^), and AOA would be operative at low oxygen to oxidize the ammonia to nitrite for anammox bacteria. The coexistence of ammonia-oxidizing archaea, ammonia-oxidizing bacteria, n-DAMO bacteria, and anammox has been reported in populations ranging from the lab scale^41^ to the natural systems^16^.

## Methods and Materials

### Soil Samples and Background

Two kinds of unconfined aquifers were selected as sampling sites: a Tianjin aquifer in China (N40°07′ E116°28′), which experiences human activity disturbance, and a Basel aquifer in Switzerland (N47°31′ E7°43′), which has little human disturbance. The Tianjin aquifer is located in the North China Plain, which plays a central role in China’s food production by supplying more than half of China’s wheat and one-third of its maize. Agricultural production in the region has grown markedly in the past decades, strongly benefiting from the fast-growing groundwater exploitation. The strong increase in the regional evapotranspiration has led to an imbalance in the groundwater budget^46^. Moreover, the nitrate pollution here is becoming another difficult issue. By contrast, the Basel aquifer, located in the Canton Basel-Landschaft of northwest Switzerland, has groundwater of drinking water quality with little to moderate human activity near the sampling site.

Depth-dependent soil cores were collected using PowerProbe (at the Tianjin site) and GeoProbe direct push drill rigs (at the Basel site) in June 2013. The aquifer of the Tianjin sampling site is between 8 and 16 m below ground level (bgl) with overlying permeable, but unsaturated, soils. Three parallel soil cores (0–20 m bgl) were collected and sectioned at 1 m intervals based on the model of PowerProbe drill rigs. The aquifer of the Basel sampling site is below 7.2 m bgl, and three parallel soil cores (0–10 m bgl) were collected and sectioned at 1.2 m intervals based on the model of GeoProbe drill rigs. The collected soil cores were sealed and transported to the laboratory at 4 °C as soon as possible. One part was immediately incubated to determine anammox activities, and another part was sieved through 0.5 mm for chemical component analysis; subsamples were stored at −80 °C for DNA extraction and subsequent molecular analysis. The groundwater samples for incubation media were also collected, and measured for dissolved oxygen (DO) concentration (Fig. 1I) and *in situ* temperatures (T, 13 ± 2 °C and 10 ± 2 °C in Tianjin and Basel aquifers, respectively), sterilized through 0.22 μm filter, and finally stored at 4 °C.

### DNA Extraction, PCR, Cloning, and Sequencing Analysis

About 0.35 g of freeze-dried soil from each sample (20 in Tianjin and 8 in Basel) was used for DNA extraction using a FastDNA SPIN Kit for Soil (Bio 101, USA), following the manufacturer’s protocol. Each sample was determined reparably three times. The extracted DNA was checked on 1% agarose gel, and the concentration was determined with Nanodrop ND-1000 ultraviolet-visible spectrophotometry (NanoDrop Technologies, Wilmington, DE, USA). Polymerase chain reaction (PCR) was performed in a C1000 thermal cycler (BioRad, USA). The function/16 S rRNA genes of anammox and related microorganism used in this study was following: hydrazine synthase β-subunit (*hzs*B) and 16 S rRNA gene of anammox bacteria, ammonia monooxygenase α-subunit (*amo*A) gene of AOB & AOA, and particulate methane monooxygenase α-subunit (*pmo*A) gene of n-DAMO. The details of primers and corresponding reaction profiles are shown in Table S1. The PCR product was gel-purified and ligated using pGEM-T Easy Vector (Promega, USA). The resulting ligation products were used to transform *Escherichia coli* JM109 competent cells, following the manufacturer’s instructions. In total, 96 clones were picked for each of the PCR products from each sampling site. PCR screens for the presence of inserts were performed using T7 and SP6 vector primers, and the amplicons were analyzed with restriction endonuclease *Hha* I, *Hae* III, and *Rsa* I (TAKARA, Dalian, China). Restriction digestion was carried out in a total volume of 20 μL that included 5 U restriction enzymes and 4 μL PCR products, and the system was incubated for 2 h at 37 °C. Digested DNA fragments were analyzed by fragment separation on a 2% (w/v) agarose gel and visualized with a GBOX/HR-E-M (Syngene, UK). Representative clones from each digestion pattern were selected for sequencing using an ABI 3730XL automated sequencer (Applied Biosystems, USA). Searches using the Basic Local Alignment Search Tool (BLAST) from the National Center for Biotechnology Information (NCBI) against the GenBank database verified that the PCR products were most closely related to the target sequences. All the sequences and their relatives obtained from the NCBI-BLAST were aligned using the Clustal X1.83 program^47^. The anammox bacterial sequences that shared 97% nucleotide similarity for 16 S rRNA and 98% nucleotide similarity for the *hzs*B gene were grouped into the same operational taxonomic unit (OTU) by employing the furthest neighbor approach using DOTUR software^48^. Biodiversity indicators (Shannon and Chao 1) were also calculated with DOTUR software. Phylogenetic trees were constructed using the neighbor-joining (NJ) mode and the maximum composite likelihood mode of the MEGA 4 software package^49^. The sequences obtained in this study are available at the NCBI under the Accession numbers of KF905049-KF905142 (anammox *hzs*B gene), GU083969-GU084010 and KF896206–KF896228 (anammox 16 S rRNA gene), KP168045–KP168078, HQ202453, and KC341050 (AOA *amo*A gene), KC341292–KC341300 (n-DAMO *pmo*A gene), and KT005575–KT005589 (AOB *amo*A gene).

### Quantitative Real-Time PCR

SYBR-Green I- based real-time qPCR assays were carried out in a volume of 20 μL containing 10 μL SYBR *Premix Ex Taq* (TAKARA, Dalian, China), 4 pmol of each primer, and 2 μL of ten-fold diluted DNA template. Each sample was determined reparably three times. Amplification and detection were carried out with an ABI Prism 7300 Sequence Detection System (Applied Biosystems, USA) with the primer set and thermal profiles compiled in Supplementary Table S1. The plasmid DNAs were extracted with a GeneJet Plasmid Miniprep Kit (Fermentas, Lithuania), and concentrations were determined on a Nanodrops ND-1000 UV-Vis Spectrophotometer (NanoDrop Technologies, Wilmington, DE, USA) for calculations of *hzs*B and *amo*A gene copy numbers. Ten-fold serial dilutions of a known copy number of the plasmid DNA were subjected to real-time qPCR in triplicate to generate an external standard curve. Melting curves were generated after each assay to check the specificity of amplification. PCR efficiencies were 90–103% (average = 92%) for the anammox bacterial *hzs*B and the archaeal and bacterial *amo*A genes. Only the results with correlation coefficients above 0.98 were used.

In the real-time qPCR quantitative assays targeting the *hzs*B gene, the detection limit of environmental samples was determined by a dilution method^18^. With the identical PCR procedure, the lowest anammox abundance was observed with the undiluted sample of CZ29-4 at ~10^3^ copies g^−1^, which was the assumed detection limit in this environmental investigation (Supplementary Fig. S2).

### Measuring rates of anammox, denitrification and nitrification with ^15^N-tracer technique

Different ^15^N- amended substrates were added for the rate determinations of various N-cycling processes. The homogenized soil samples with known weight and bulk density were transferred to helium-flushed, 12-mL glass vials (Exetainer, Labco, UK) together with *in situ* media water at *in situ* soil temperature. Triplicate activity tests were performed for each soil sample. For anammox and denitrification, the resulting slurries were pre-incubated under anoxic conditions to remove residual nitrite/nitrate (NO_x_^−^) in soils and incubation media according to the established protocol^17^. It is emphasized that, because of the different soil sample backgrounds, the pre-incubated times to remove the residual NO_x_^−^ varied in a wide range, from 45 h to several days, in order to ensure completely anoxic conditions in the soils slurries, which was important for the appropriate controls. Subsequently, 100 μL of N_2_-purged stock solution of each isotopic mixture was added to obtain a final concentration of about 10% of the initial content background to avoid overestimate. The isotopic mixtures were as follows: (1) ^15^NH_4_^+^ (^15^N at 99.6%, negative control), (2) ^15^NH_4_^+^ + ^14^NO_3_^−^ (positive control) and (3) ^15^NO_3_^−^ (^15^N at 99%, for calculations). Incubation of the slurries was stopped at 0 h, 3 h, 6 h, 12 h, 24 h by 200 μL 7 M ZnCl_2_ injected. The slurries amended solely with ^15^NH_4_^+^ showed no significant accumulation of ^15^N_2_-labeled gas (^29^N_2_ and/or ^30^N_2_) in any sample, indicating that all ambient ^14^NO_x_^−^ had been consumed during the pre-incubations. When both ^15^NH_4_^+^ and ^14^NO_3_^−^ were added, ^29^N_2_ accumulated in every soil sample with no accumulation of ^30^N_2_. This pattern was reproducible and indicated that the anammox process was detectable in the soils. Slurries amended solely with ^15^NO_3_^−^ showed significant anammox and denitrification rates. For nitrification process, the rate was measured as the production of ^15^NO_2_^−^ from incubations with ^15^NH_4_^+^ (100 μL injection) via the reduction of NO_2_^−^ by sulfuric acid to N_2_ chemically. A 12 h or longer incubation was needed to achieve better NO_2_^−^ reduction efficiency, as described by ref. 50. All samples were incubated with ^15^N or ^14^N compounds to a final concentration that corresponded to a maximum of 10% of the *in situ* concentrations. The possibility of overestimating the potential rate measurements was therefore minimized. Three parallel assays were conducted for each soil sample. Process rates were calculated from produced ^29^N_2_ or ^30^N_2_ as measured by gas chromatography-isotope ratio mass spectrometers (GasBench-II, Elemental Analyzer FLASH 2000 HT, MAT 253, Germany).

### Analytical procedures of environmental variables

The environmental variables of soils, including NH_4_^+^, NO_x_^−^, total nitrogen (TN), total phosphorus (TP), total organic matter (TOM), pH, and moisture content, were investigated^51^. A mixture of 5 g of sieved fresh soil sample and 12.5 mL of ddH_2_O (a soil-to-water ratio of 1:2.5) was measured for pH value. Both NH_4_^+^-N and NO_x_^−^-N were extracted from 6 g of sieved soil by shaking for 1 h at room temperature with 30 mL 2 M KCl, and were then measured spectrophotometrically. The TN was determined by potassium persulfate digestion and measured spectrophotometrically. The TOM was measured using the potassium dichromate method. The DO and T in groundwater were monitored *in-situ* using a WTW oxi/340i oxygen probe (WTW Company, Weilheim, Germany). Triplicates were run for quality assurance (QA).

### Statistical analysis

The statistical analyses were conducted using SPSS Statistics 18.0 software (Predictive Analytics Software Statistics). The correlations between various variables were computed by Spearman correlation analysis. Multiple linear regressions (stepwise regression) were used to identify the most determining variables for the dependent variables. Unless otherwise specified, the level of significance in this study was α ≤ 0.05.

## Additional Information

**How to cite this article**: Wang, S. *et al*. Nitrogen loss by anaerobic ammonium oxidation in unconfined aquifers soils. *Sci. Rep.* **7**, 40173; doi: 10.1038/srep40173 (2017).

**Publisher's note:** Springer Nature remains neutral with regard to jurisdictional claims in published maps and institutional affiliations.

## Supplementary Material

Supplementary Information

## References

[b1] Falkowski P. G., Fenchel T. & Delong E. F. The microbial engines that drive earth’s biogeochemical cycles. Science 320, 1034–1039 (2008). 18497287 10.1126/science.1153213

[b2] Francis C. A., Beman J. M. & Kuypers M. M. New processes and players in the nitrogen cycle: the microbial ecology of anaerobic and archaeal ammonia oxidation. ISME J. 1, 19–27 (2007). 18043610 10.1038/ismej.2007.8

[b3] Jetten M. S. . The anaerobic oxidation of ammonium. FEMS Microbiol. Rev. 22, 421–437 (1998). 9990725 10.1111/j.1574-6976.1998.tb00379.x

[b4] Canfield D. E., Glazer A. N. & Falkowski P. G. The evolution and future of earth’s nitrogen cycle. Science 330, 192–196 (2010). 20929768 10.1126/science.1186120

[b5] Galloway J. N. . Nitrogen cycles: past, present, and future. Biogeochemistry 70, 153–226 (2004).

[b6] Burgin A. J. & Hamilton S. K. Have we overemphasized the role of denitrification in aquatic ecosystems? A review of nitrate removal pathways. Front Ecol. Environ. 5, 89–96 (2007).

[b7] Dalsgaard T., Canfield D. E., Petersen J., Thamdrup B. & Acuňa-González J. N_2_ production by the anammox reaction in the anoxic water of Golfo Dulce, Costa Rica. Nature 422, 606–608 (2003). 12686998 10.1038/nature01526

[b8] Kuypers M. M. M. . Anaerobic ammonium oxidation by anammox bacteria in Black Sea. Nature 422, 608–611 (2003). 12686999 10.1038/nature01472

[b9] Kartal B. . Molecular mechanism of anaerobic ammonium oxidation. Nature 479, 127–130 (2011). 21964329 10.1038/nature10453

[b10] Arrigo K. R. Marine microorganisms and global nutrient cycles. Nature 437, 349–355 (2005). 16163345 10.1038/nature04159

[b11] Schmid M. C. . Anaerobic ammonium-oxidizing bacteria in marine environments: widespread occurrence but low diversity. Environ. Microbiol. 9, 1476–1484 (2007). 17504485 10.1111/j.1462-2920.2007.01266.x

[b12] Kuypers M. M. M. . Massive nitrogen loss from the Benguela upwelling system through anaerobic ammonium oxidation. Proc. Natl. Acad. Sci. USA 102, 6478–6483 (2005). 15843458 10.1073/pnas.0502088102 PMC556276

[b13] Lam P. . Linking crenarchaeal and bacterial nitrification to anammox in the Black Sea. Proc. Natl. Acad. Sci. USA 102, 6478–6483 (2007). 10.1073/pnas.0611081104 PMC1849958 17420469

[b14] Lam P. . Revising the nitrogen cycle in the Peruvian oxygen minimum zone. Proc. Natl. Acad. Sci. USA 106, 4752–4757 (2009). 19255441 10.1073/pnas.0812444106 PMC2649953

[b15] Nie S. . Nitrogen loss by anaerobic oxidation ammonium in rice rhizosphere. ISME J. 9, 2059–67 (2015). 25689022 10.1038/ismej.2015.25 PMC4542037

[b16] Wang S., Zhu G., Peng Y., Jetten M. S. M. & Yin C. Anammox bacterial abundance, activity and contribution in riparian sediments of the Pearl River Estuary. Environ. Sci. Technol. 46, 8834–8842 (2012). 22816681 10.1021/es3017446

[b17] Zhu G. . Hotspots of anaerobic ammonia oxidation at land-freshwater interfaces. Nature Geosci. 6, 103–107 (2013).

[b18] Zhu G. . Ubiquitous anaerobic ammonium oxidation in inland waters: an overlooked nitrous oxide mitigation process. Sci. Rep. 5, doi: 10.1038/srep17306 (2015). PMC4661425 26610807

[b19] Humbert S. . Molecular detection of anammox bacteria in terrestrial ecosystems: distribution and diversity. ISME J. 4, 450–454 (2010). 20010634 10.1038/ismej.2009.125

[b20] Zhu G., Jetten M. S. M., Kuschk P., Ettwig K. & Yin C. Potential roles of anaerobic ammonia and methane oxidation in the nitrogen cycle of wetland ecosystems. Appl. Microbiol. Biotechnol. 86, 1043–1055 (2010). 20195861 10.1007/s00253-010-2451-4

[b21] Zhu G. . Anaerobic ammonia oxidation in a fertilized paddy soil. ISME J. 5, 1905–1912 (2011b). 21593796 10.1038/ismej.2011.63 PMC3223303

[b22] Aeschbach-Hertig W. & Gleeson T. Regional strategies for the accelerating global problem of groundwater depletion. Nat. Geosci. 5, 853–861 (2012).

[b23] Taylor R. G. . Ground water and climate change. Nat. Clim. Chang. 3, 322–329 (2013).

[b24] Böhlke J. K., Smith R. L. & Miller D. N. Ammonium transport and reaction in contaminated groundwater: Application of isotope tracers and isotope fractionation studies. Water Resour. Res. 42, 1–19 (2006).

[b25] Smits T. H. M., Huttmann A., Lerner D. N. & Holliger C. Detection and quantification of bacteria involved in aerobic and anaerobic ammonium oxidation in an ammonium-contaminated aquifer. Biorem. J. 13, 41–51 (2009).

[b26] Clark I. . Origin and fate of industrial ammonium in anoxic ground water—^15^N evidence for anaerobic oxidation (Anammox). Ground Water Monit. R. 28, 73–82 (2008).

[b27] Smith R. L., Böhlke J. K., Song B. & Tobias C. Role of anaerobic ammonium oxidation (anammox) in nitrogen removal from a freshwater aquifer. Environ. Sci. Technol. 49, 12169–77 (2015). 26401911 10.1021/acs.est.5b02488

[b28] Ettwig K. F. . Nitrite-driven anaerobic methane oxidation by oxygenic bacteria. Nature 456, 543–560 (2010). 10.1038/nature08883 20336137

[b29] Strous M. . Missing lithotroph identified as new planctomycete. Nature 400, 446–449 (1999). 10440372 10.1038/22749

[b30] Moore T. A. . Prevalence of anaerobic ammonia-oxidizing bacteria in contaminated groundwater. Environ. Sci. Technol. 45, 7217–7225 (2011). 21786759 10.1021/es201243t

[b31] Smith R. L. Is anaerobic ammonium oxidation (“anammox”) important for nitrogen cycling in groundwater? 125^th^ Anniversary Annual Meeting & Expo (2013).

[b32] Jewell T. N. M. . Metatranscriptomic analysis of groundwater reveals an active anammox bacterial population. AGU Fall Meeting Abstracts (2014).

[b33] Schubert C. J. . Anaerobic ammonium oxidation in a tropical freshwater system (Lake Tanganyika). Environ. Microbiol. 8, 1857–63 (2006). 16958766 10.1111/j.1462-2920.2006.01074.x

[b34] Wang S. . Anaerobic ammonium oxidation in traditional municipal wastewater treatment plants with low-strength ammonium loading: widespread but overlooked. Water Res. 84, 66–75 (2015). 26210031 10.1016/j.watres.2015.07.005

[b35] Hu B. L. . New anaerobic, ammonium-oxidizing community enriched from peat soil. Appl. Environ. Microbiol. 77, 966–71 (2011). 21148690 10.1128/AEM.02402-10 PMC3028707

[b36] Hu B. . Enrichment of an anammox bacterial community from a flooded paddy soil. Environ. Microbiol. Rep. 5, 483–489 (2013). 23754729 10.1111/1758-2229.12038

[b37] Rivett M. O., Buss S. R., Morgan P., Smith J. W. & Bemment C. D. Nitrate attenuation in groundwater: a review of biogeochemical controlling processes. Water Res. 42, 4215–4232 (2008). 18721996 10.1016/j.watres.2008.07.020

[b38] Schmidt S. I. & Jürgen Hahn H. What is groundwater and what does this mean to fauna? Limnologica 42, 1–6 (2012).

[b39] Zhu G. . Biological removal of nitrogen from wastewater. In Reviews of environmental contamination and toxicology Springer, New York, 159–195 (2008). 18020306 10.1007/978-0-387-71724-1_5

[b40] Kartal B. . Anammox-growth physiology, cell biology, and metabolism. Adv. Microb. Physiol. 60, 211–262 (2012). 22633060 10.1016/B978-0-12-398264-3.00003-6

[b41] Yan J. . Mimicking the oxygen minimum zones: stimulating interaction of aerobic archaeal and anaerobic bacterial ammonia oxidizers in a laboratory-scale model system. Environ. Microbiol. 14, 3146–3158 (2012). 23057688 10.1111/j.1462-2920.2012.02894.x PMC3558802

[b42] Woebken D., Fuchs B. M., Kuypers M. M. M. & Amann R. Potential Interactions of particle-associated anammox bacteria with bacterial and archaeal partners in the Namibian Upwelling System. Appl. Environ. Microbiol. 73, 4648–4657 (2007). 17526789 10.1128/AEM.02774-06 PMC1932835

[b43] Luesken F. A. . Simultaneous nitrite-dependent anaerobic methane and ammonium oxidation processes. Appl. Environ. Microbiol. 77, 6802–6807 (2011). 21841030 10.1128/AEM.05539-11 PMC3187098

[b44] Shi Y. . Nitrogen removal from wastewater by coupling anammox and methane-dependent denitrification in a membrane biofilm reactor. Environ. Sci. Technol. 47, 11577–83 (2013). 24033254 10.1021/es402775z

[b45] Martens-Habbena W., Berube P. M., Urakawa H., de la Torre J. R. & Stahl D. A. Ammonia oxidation kinetics determine niche separation of nitrifying Archaea and Bacteria. Nature 461, 976–979 (2009). 19794413 10.1038/nature08465

[b46] Gleeson T., Wada Y., Bierkens M. F. P. & van Beek L. P. H. Water balance of global aquifers revealed by groundwater footprint. Nature 488, 197–200 (2012). 22874965 10.1038/nature11295

[b47] Thompson J. D., Gibson T. J., Plewniak F., Jeanmougin F. & Higgins D. G. The CLUSTALX windows interface: flexible strategies for multiple sequence alignment aided by quality analysis tools. Nucleic Acids Res 25, 4876–4882 (1997). 9396791 10.1093/nar/25.24.4876 PMC147148

[b48] Schloss P. D. & Handelsman J. Introducing DOTUR, a computer program for defining operational taxonomic units and estimating species richness. Appl. Environ. Microbiol. 71, 1501–1506 (2005). 15746353 10.1128/AEM.71.3.1501-1506.2005 PMC1065144

[b49] Tamura K. . MEGA5: molecular evolutionary genetics analysis using maximum likelihood, evolutionary distance, and maximum parsimony methods. Mol. Biol. Evol. 28, 2731–2739 (2011). 21546353 10.1093/molbev/msr121 PMC3203626

[b50] Füssel J. . Nitrite oxidation in the Namibian oxygen minimum zone. ISME J. 6, 1200–1209 (2012). 22170426 10.1038/ismej.2011.178 PMC3358024

[b51] Bao S. D. Chemical Analysis for Agricultural Soil China Agriculture Press, Beijing (2000).

