# Supplementary material for: Nitrogen loss by anaerobic ammonium oxidation in unconfined aquifer soils
Source: Sci Rep. 2017 Jan 10;7:40173. doi: 10.1038/srep40173 (PMC5223210; doi:10.1038/srep40173)
Supplement: Supplementary Information [file srep40173-s1.pdf]

Nitrogen loss by anaerobic ammonium oxidation in unconfined aquifer soils

Shanyun Wang[1], Dirk Radny[2], Shuangbing Huang[3], Linjie Zhuang[1], Siyan Zhao[1], Michael Berg[2], Mike S. M. Jetten[4], Guibing Zhu[1, 5 *]

1. Key Laboratory of Drinking Water Science and Technology, Research Center for Eco-Environmental Sciences, Chinese Academy of Sciences, Beijing, China
2. Department of Water Resources and Drinking Water, Swiss Federal Institute of Aquatic Science and Technology, Switzerland
3. Institute of Hydrogeology and Environmental Geology, Chinese Academy of Geological Sciences, Shijiazhuang 050061, China
4. Department of Microbiology, Radboud University, Nijmegen, Netherlands
5. Department of Biogeochemistry, Max Planck Institute for Marine Microbiology, Bremen, Germany

* Corresponding author, E-mail: gbzhu@rcees.ac.cn and gzhu@mpi-bremen.de

Key Words: anaerobic ammonium oxidation; aquifer soils; biogeochemistry; nitrogen loss;

Supplementary information

Summary

We provide here supplementary materials such as figures, tables, complementary data, and experimental analysis to illustrate the main text as followed:

Supplementary figures and tables

Supplementary Figure S1 The *in situ* photos at the sampling site in Tianjin, China.

Supplementary Figure S2 Quantitative PCR plots of the standard curve (***a***), the slope was -3.42, and R^2 was 0.99198, the amplification plots of standards samples, negative control, and environmental sample of detection limit (***b***), the melting curves of the standards samples (***c***) and the melting curve of detection limit sample CZ29-4 (***d***)

Supplementary Figure S3 Examples of concentrations of $^{29}N_2$ and $^{30}N_2$ in samples amended with ^{15}N labeled on ammonia and nitrate, separately. Case A and case B serve as negative and positive controls, respectively.

Supplementary Figure S4 The affiliation of AOA&AOB *amo*A gene and n-DAMO *pmo*A gene sequences, retrieved from deeper soils (15 m bgl) of the Tianjin aquifer. The detailed information of AOA, AOB, and n-Damo bacteria clones sequences of each OTU and accession numbers in Genbank are listed in Supplementary Table S6.

Supplementary Table S1 The correlation of anammox rate, abundance of the *hzs*B gene, and chemical characteristics in Tianjin soil cores (n=20)

Supplementary Table S2 The correlation of anammox rate, abundance of *hzsb* gene and chemical characteristics in Basel soil cores (n=8)

Supplementary Table S3 The layout of OTU number and related anammox bacterial *hzsB* gene sequence accession numbers in the tree

Supplementary Table S4 The layout of OTU number and related anammox bacterial 16S rRNA sequence accession numbers in the tree

Supplementary Table S5 Results of multiple linear stepwise regression analysis on nitrification activity in Tianjin soil cores (n=20)

Supplementary Table S6 The layout of OTU number and related ammonia-oxidizing archaeon *amo*A gene, ammonia-oxidizing bacterium *amo*A gene, and *pmo*A gene sequence accession numbers in the tree

Supplementary Table S7 Primers used in this study and corresponding reaction profiles

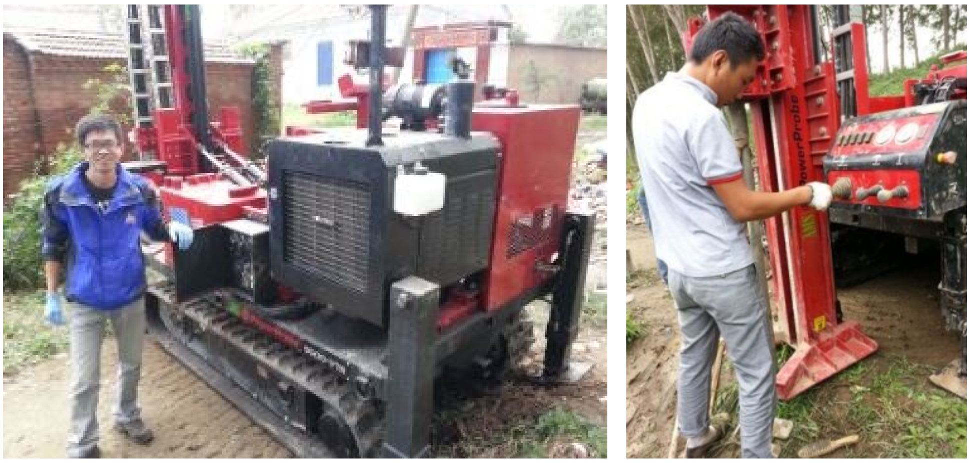

Supplementary Figure S1 The *in situ* photos at the sampling site in Tianjin, China. The person in left photo is Chao Xia, and right is Linjie Zhuang.

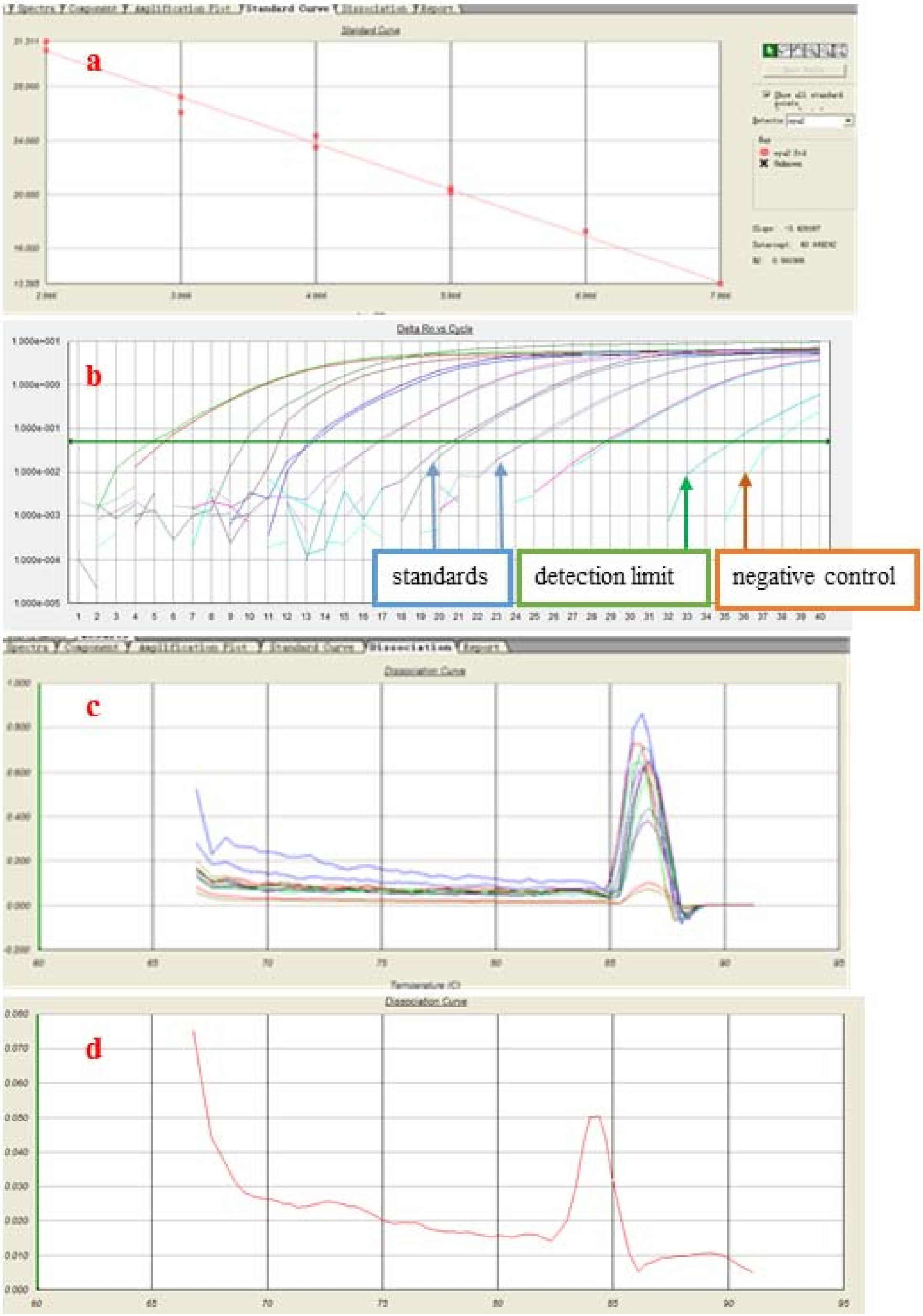

Supplementary Figure S2 Quantitative PCR plots of the standard curve (***a***), the slope was -3.42, and R^2 was 0.99198, the amplification plots of standards samples, negative control, and environmental sample of detection limit (***b***), the melting curves of the standards samples (***c***) and the melting curve of detection limit sample CZ29-4 (***d***)

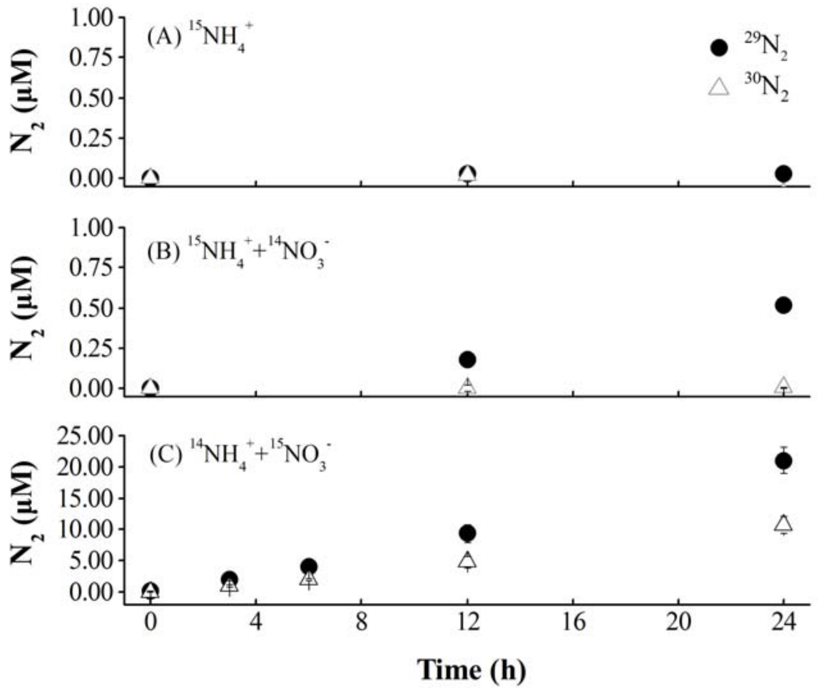

Supplementary Figure S3 Examples of concentrations of $^{29}N_2$ and $^{30}N_2$ in samples amended with ^{15}N labeled on ammonia and nitrate, separately. Case A and case B serve as negative and positive controls, respectively.

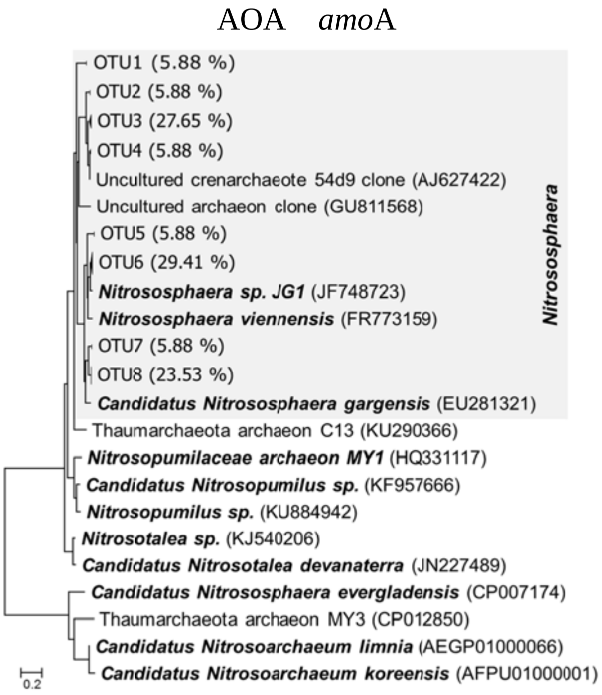

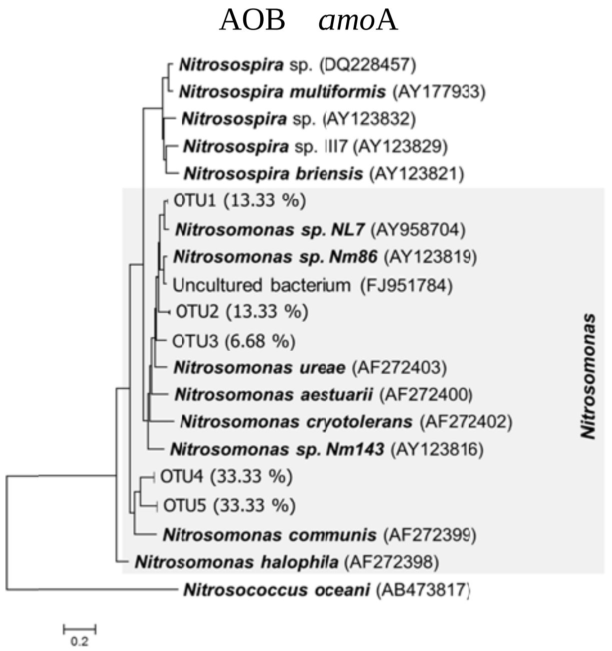

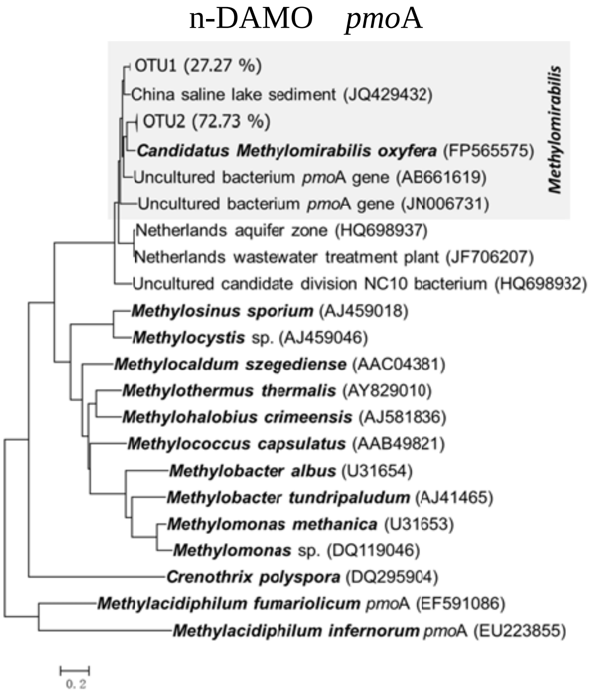

Supplementary Figure S4 The affiliation of AOA&AOB *amo*A gene and n-DAMO *pmo*A gene sequences, retrieved from deeper soils (15 m bgl) of the Tianjin aquifer. The detailed information of AOA, AOB, and n-Damo bacteria clones sequences of each OTU and accession numbers in Genbank are listed in Supplementary Table S6.

Supplementary Table S1 The correlation of anammox rate, abundance of the *hzs*B gene, and chemical characteristics in Tianjin soil cores (n=20)

		NH_4^+	NO_x^-	TN	TP	TOM	Water Content	pH	Abundance *hzs*B	Anammox rate
Abundance *hzs*B	R^2	0.113	0.425	-0.515*	-0.535*	-0.430	0.957**	-0.520*	1	0.862**
	p	0.636	0.062	0.020	0.015	0.058	0.000	0.019		0.000
Anammox rate	R^2	0.120	0.423	-0.433	-0.378	-0.345	0.844**	-0.409	0.862**	1
	p	0.615	0.063	0.057	0.100	0.136	0.000	0.073	0.000	

**. Correlation is significant at the 0.01 level (2-tailed).

*. Correlation is significant at the 0.05 level (2-tailed).

Supplementary Table S2 The correlation of anammox rate, abundance of *hzs*B gene and chemical characteristics in Basel soil cores (n=8)

		NH_4^+	NO_x^-	TN	TOM	Water Content	pH	Abundance *hzs*B	Anammox rate
Abundance *hzs*B	R^2	-0.277	-0.279	-0.311	-0.629	0.828*	0.395	1	1.000**
	p	0.507	0.504	0.453	0.095	0.011	0.332		0.000
Anammox rate	R^2	-0.260	-0.272	-0.311	-0.629	0.826*	0.409	1.000**	1
	p	0.533	0.514	0.453	0.095	0.012	0.314	0.000	

*. Correlation is significant at the 0.05 level (2-tailed).

**. Correlation is significant at the 0.01 level (2-tailed).

Supplementary Table S3 The layout of OTU number and related anammox bacterial *hzs*B gene sequence accession numbers in the tree

OTU	Sequence number	Sequence ID		Accession number(s)
		Tianjin aquifer	Basel aquifer	
1#	34		NC12-11, NC12-9, NC12-51, NC15-33, NC12-4, NC12-14, NC15-38, NC15-34, NC15-5, NC15-31, NC12-27, NC15-48,NC15-6, NC12-42, NC12-5, NC15-2, NC12-13, NC15-3, NC15-41, NC15-30, NC15-9, NC12-16, NC15-29, NC12-3, NC12-2, NC12-12, NC12-39, NC12-17, NC12-15, NC12-35, NC12-52, NC15-47, NC12-32, NC15-28	KF905061, KF905062, KF905093, KF905085, KF905066, KF905070, KF905081, KF905084, KF905055, KF905087, KF905098, KF905072, KF905054, KF905094, KF905063, KF905058, KF905071, KF905057, KF905078, KF905088, KF905051, KF905067, KF905100, KF905064, KF905065, KF905060, KF905095, KF905059, KF905069, KF905096, KF905092, KF905099, KF905097, KF905089
2#	2		NC15-11, NC15-32	KF905049, KF905086
3#	6		NC15-8, NC15-39, NC15-7, NC15-43, NC15-40, NC15-36	KF905052, KF905080, KF905053, KF905076, KF905079, KF905083
4#	1		NC15-4	KF905056
5#	1		NC15-10	KF905050
6#	1		NC15-26	KF905090
7#	1		NC15-25	KF905091
8#	1	NCsur-19		KF905114
9#	14	NCsur-12, NCsur-15, NCsur-72, NCsur-65, NCsur-24, NCsur-8, NCsur-40, NCsur-42, NCsur-38, NCsur-46, NCsur-25,NCsur-13, NCsur-1	NC15-46	KF905109, KF905118, KF905123, KF905127, KF905111, KF905120, KF905138, KF905137, KF905140, KF905136, KF905107, KF905119, KF905110, KF905073
10#	1		NC15-1	KF905068
11#	3		NC15-45, NC15-42, NC15-27	KF905074, KF905077, KF905101
12#	2		NC15-44, NC15-37	KF905075, KF905082
13#	1	NCsur-68		KF905125
14#	3	NCsur-17, NCsur-16, NCsur-23		KF905116, KF905117, KF905112
15#	21	NCsur-36, NCsur-26, NCsur-70, NCsur-52, NCsur-69, NCsur-5, NCsur-28, NCsur-60, NCsur-49, NCsur-48, NCsur-50,NCsur-14, NCsur-18, NCsur-31, NCsur-3, NCsur-66,NCsur-37, NCsur-39, NCsur-59, NCsur-53, NCsur-64		KF905102, KF905106, KF905124, KF905132, KF905142, KF905121, KF905105, KF905129, KF905134, KF905135, KF905133, KF905108, KF905115, KF905103, KF905122, KF905126, KF905141, KF905139, KF905130, KF905131, KF905128
16#	2	NCsur-30, NCsur-22		KF905104, KF905113

Supplementary Table S4 The layout of OTU number and related anammox bacterial 16S rRNA sequence accession numbers in the tree

OTU	Sequence number	Sequence ID		Accession number(s)
		Tianjin aquifer	Basel aquifer	
1#	18		NC6-1, NC6-9, NC6-54, NC6-61, NC6-2, NC6-3, NC6-4, NC6-6, NC6-12, NC6-8, NC6-13, NC6-15, NC6-45, NC6-10, NC6-100, NC6-36, NC7-3, NC7-103	GU083968, GU083976, GU083991, GU083973, GU083969, GU083970, GU083971, GU083993, GU083992, GU083997, GU083995, GU083982, GU083988, GU083977, GU083994, GU083990, GU083999, GU084009
2#	10	BYHE-22, BYHE-12, BYHE-8, BYHE-3, BYHE-18, BYHE-20, BYHE-21	NC6-48, NC6-81, NC6-7	KF896208, KF896217, KF896221, KF896226 KF896212 KF896210 KF896209 GU083996, GU083975, GU083974
3#	21		NC6-112, NC7-15, NC6-20, NC7-107, NC7-20, NC7-97, NC6-113, NC7-34, NC7-8, NC7-18, NC6-14, NC6-16, NC6-17, NC7-2, NC7-105, NC6-11, NC7-108, NC7-28, NC6-5, NC6-107, NC6-19	GU083979, GU083998, GU083987, GU084008, GU084000, GU084007, GU083980, GU084004, GU084002, GU084003, GU083981, GU083983, GU083984, GU084005, GU084006, GU083978, GU084001, GU084010, GU083972, GU083989, GU083986
4#	16	BYHE-24, BYHE-14, BYHE-19, BYHE-10, BYHE-16, BYHE-6, BYHE-9, BYHE-5, BYHE-2, BYHE-1, BYHE-23, BYHE-7, BYHE-17, BYHE-13, BYHE-11, BYHE-4		KF896206, KF896215, KF896211, KF896219, KF896214, KF896223, KF896220, KF896224, KF896227, KF896228, KF896207, KF896222, KF896213, KF896216, KF896218, KF896225
5#	1		NC6-18	GU083985

Supplementary Table S5 Results of multiple linear stepwise regression analysis on nitrification activity in Tianjin soil cores (n=20)

Results of the model	Adjusted R^2	*p*-value of the model	variables	Adjusted R^2	*p*-value of coefficient
Nitrification activity = 362.3 + 3.2×10^{-7} AOB - 44.8 pH	0.805	0.000	AOB	0.598	0.000
			pH	0.207	0.000
			Intercept		0.000

All variables left in the model are significant at the 0.050 level.

AOB: The abundance of AOB.

Supplementary Table S6 The layout of OTU number and related ammonia-oxidizing archaeon *amo*A gene, ammonia-oxidizing bacterium *amo*A gene, and nitrite-dependent anaerobic methane oxidation *pmo*A gene sequence accession numbers in the tree

	OTU	Sequence number	Sequence ID	Accession number(s)
AOA (*amo*A gene)	1#	2	NC-15-2-5, NC-15-6-2	KP168071, KP168074
	2#	2	NC-15-2-9, NC-15-6-3	KP168072, KP168075
	3#	6	NC-15-48, NC-15-40, NC-15-2-1, NC-15-6-15, NC-15-2-2, NC-15-6-14	HQ202453, KP168065, KP168069, KP168078, KP168068, KP168077,
	4#	2	NC-15-2-3, NC-15-6-1	KP168070, KP168073
	5#	2	NC-15-42, NC-15-43	KP168052, KP168064
	6#	10	NC-15-16, NC-15-73, NC-15-17, NC-15-38, NC-15-90, NC-15-14, NC-15-47, NC-15-15, NC-15-5, NC-15-59	KP168045, KP168055, KP168057, KP168062, KP168056, KP168051, KP168047, KP168062, KC341050, KP168061
	7#	2	NC-15-2-4, NC-15-6-13	KP168067, KP168173
	8#		NC-15-3, NC-15-88, NC-15-29, NC-15-87, NC-15-2, NC-15-66, NC-15-67, NC-15-39	KP168049, KP168058, KP168059, KP168046, KP168048, KP168054, KP168066, KP168060
DAMO (*pmo*A gene)	1#	3	NC-12-31,NC-12-33,NC-12-40	KC341298, KC341299, KC341300
	2#	8	NC-15-02, NC-15-05, NC-15-12, NC-15-04, NC-15-01, NC-15-08, NC-15-07, NC-15-11	KC341293, KC341295, KC341297, KC341294, KC341292, KC341291, KC341290, KC341296
AOB (*amo*A gene)	1#	2	AOB-11, AOB-14	KT005585, KT005586
	2#	2	AOB-12, AOB-13	KT005587, KT005588
	3#	1	AOB-15	KT005589
	4#	5	AOB-1, AOB-3, AOB-4, AOB-2, AOB-5	KT005584, KT005580, KT005581, KT005582, KT005583
	5#	5	AOB-6, AOB-7, AOB-10, AOB-8, AOB-9	KT005575, KT005576, KT005579, KT005577, KT005578

Supplementary Table S7 Primers used in this study and corresponding reaction profiles

Specificity	Primer	Sequence (5'-3')	Thermal profiles	Reference
Planctomycetales (PCR)	pla46f	GGATTAGGCATGCAAGTC	5 min at 94°C, 30 cycles consisting of 1 min at 94°C, 1 min at 50°C and 2 min at 72°C	1-2
	630r	CAKAAAGGAGGTGATCC		
Anammox 16S rRNA (PCR)	Amx368f	TTCGCAATGCCCGAAAGG	10 min at 96°C, 30 cycles consisting of 1 min at 96°C, 1 min at 52°C and 1 min at 72°C	3
	Amx820r	AAAACCCCTCTACTTAGTGCCC		
Anammox *hzs*B (qPCR)	HSBeta396F	ARGGHTGGGGHAGYTGGAAG	3 min at 95°C, 40 cycles consisting of 30 s at 95°C, 30 s at 59°C and 30 s at 72°C	4
	HSBeta742R	GTYCCHACRTCATGVGTCTG		
AOA *amo*A ((q)PCR)	archea-*amo*AF	STAATGGTCTGGCTTAGACG	5 min at 95°C , 35 cycles consisting of 45 s at 94°C, 1 min at 53°C and 1 min at 72°C	5
	archea-*amo*AR	GCGGCCATCCATCTGTATGT		
AOB *amo*A ((q)PCR)	*amo*A-1F	GGGGTTTCTACTGGTGGT	5 min at 95°C , 35 cycles consisting of 30 s at 94°C, 30 s at 55°C and 1 min at 72°C	6
	*amo*A-2R	CCCCTCKGSAAAGCCTTCTTC		
damo *pmo*A (PCR)	A189F	GGNGACTGGGACTTYTGG	3 min at 95°C, followed by 40 cycles of 30 s at 95°C, 30 s at 63°C and 30 s at 72°C	7
	cmo682	AAAYCCGGCRAAGAACGA		
damo *pmo*A (PCR)	cmo182	TCACGTTGACGCCGATCC	10 min at 95°C, followed by 30 cycles of 60 s at 95°C, 60 s at 59°C and 60 s at 72°C	
	cmo568	GCACATACCCATCCCCATC		

1. Juretschko, S., Timmermann, G., Schmid, M., Schleifer, K. H., Pommerening-Röser, A., Koops, H. P., Wagner, M. Combined molecular and conventional analyses of nitrifying bacterium diversity in activated sludge: *Nitrosococcus mobilis* and *Nitrospira*-like bacteria as dominant populations. *Appl. Environ. Microbiol.* **64(8),** 3042-3051 (1998).
2. Neef, A., Amann, R., Schlesner, H. & Schleifer, K. H. Monitoring a widespread bacterial group: in situ detection of planctomycetes with 16S rRNA-targeted probes. *Microbiology* **144,** 3257-3266 (1998).
3. Schmid, M. C., Maas, B., Dapena, A., de Pas-Schoonen, K. V., de Vossenberg, J. V., Kartal, B., *et al.* Biomarkers for in situ detection of anaerobic ammonium-oxidizing (anammox) bacteria. *Appl. Environ. Microbiol.* **71,** 1677-1684 (2005).
4. Wang, Y., Zhu, G., Harhangi, H. R., Zhu, B., Jetten, M. S. M., Yin, C., *et al.* Co-occurrence and distribution of nitrite-dependent anaerobic ammonium and methane-oxidizing bacteria in a paddy soil. *FEMS Microbiol. Lett.* **336,** 79-88 (2012).
5. Francis, C. A., Roberts, K. J., Beman, J. M., Santoro, A. E. & Oakley, B. B. Ubiquity and diversity of ammonia-oxidizing archaea in water columns and sediments of the ocean. *Proc. Natl. Acad. Sci. USA* **102,** 14683-14688 (2005).
6. Wang, S., Wang, Y., Feng, X., Zhai, L. & Zhu, G. Quantitative analyses of ammonia-oxidizing archaea and bacteria in the sediments of four nitrogen-rich wetlands in China. *Appl. Microbiol. Biotechnol.* **90,** 779-787 (2011).
7. Luesken, F. A., Zhu, B., van Alen, T. A., Butler, M. K., Diaz, M. R., Song, B., *et al.* *pmo*A primers for detection of anaerobic methanotrophs. *Appl. Environ. Microbiol.* **77,** 3877-3880 (2011).
